# Alice in Wonderland Syndrome: A Clinical and Pathophysiological Review

**DOI:** 10.1155/2016/8243145

**Published:** 2016-12-27

**Authors:** Giulio Mastria, Valentina Mancini, Alessandro Viganò, Vittorio Di Piero

**Affiliations:** ^1^Department of Neurology and Psychiatry, Sapienza University of Rome, Rome, Italy; ^2^Department of Anatomy, Histology, Forensic Medicine and Orthopaedics, Sapienza University of Rome, Rome, Italy; ^3^University Consortium for Adaptive Disorders and Head Pain (UCADH), Pavia, Italy

## Abstract

Alice in Wonderland Syndrome (AIWS) is a perceptual disorder, principally involving visual and somesthetic integration, firstly reported by Todd, on the literary suggestion of the strange experiences described by Lewis Carroll in Alice in Wonderland books. Symptoms may comprise among others aschematia and dysmetropsia. This syndrome has many different etiologies; however EBV infection is the most common cause in children, while migraine affects more commonly adults. Many data support a strict relationship between migraine and AIWS, which could be considered in many patients as an aura or a migraine equivalent, particularly in children. Nevertheless, AIWS seems to have anatomical correlates. According to neuroimaging, temporoparietal-occipital carrefour (TPO-C) is a key region for developing many of AIWS symptoms. The final part of this review aims to find the relationship between AIWS symptoms, presenting a pathophysiological model. In brief, AIWS symptoms depend on an alteration of TPO-C where visual-spatial and somatosensory information are integrated. Alterations in these brain regions may cause the cooccurrence of dysmetropsia and disorders of body schema. In our opinion, the association of other symptoms reported in literature could vary depending on different etiologies and the lack of clear diagnostic criteria.

## 1. Introduction

Alice in Wonderland Syndrome (AIWS) is a rare perceptual disorder, chiefly affecting the integration mechanisms among sensory associative cortices that are involved in the development of internal-external relationship. Cardinal alteration of AIWS is the unbalance between the self-representation and the external world, so that patients with AIWS may have an erroneous perception of their body size with respect to the external environment or a rework of the external space to their own body reference.

AIWS remains a poorly known and probably misdiagnosed syndrome. This variableness in the diagnostic process is due to the fact that no univocally accepted diagnostic criteria for this disease have been made. AIWS can occur at any age but mostly in children and it is not solely related to one medical condition but rather can have several causes. However, a link with migraine seems to be suggested by the high frequency of cooccurrence of the two diseases.

In this review, we will discuss the original description of perceptual alterations by Lewis Carroll and will frame them into the main clinical features of AIWS as presented in several case reports. A main topic will be the critical review of available classifications. Since no clear pathophysiological mechanism for AIWS is known, an anatomical study of correlation will be presented considering all cases in which neuroimaging data were available.

## 2. Method

A literature review was carried out in PubMed using “AIWS” and “Alice in Wonderland Syndrome” as key words that produced 148 results. Two independent researchers reviewed all abstracts and, among these 148 articles, 94 were collected for further evaluation because they reported relevant information about the syndrome. Disagreements were resolved by consensus. In order to collect data for pathophysiological sections, we consulted PubMed indexed articles using “macropsia”, “micropsia”, “macrosomatognosia”, “microsomatognosia”, etc., even if the term Alice in Wonderland Syndrome was not expressly used. However, to be included in the review, case descriptions had to be suggestive of AIWS as by judgment of two independent researchers. Other articles that were not indexed in PubMed were found by consulting the references included in selected publications. Other papers were selected according to reviewers' suggestions.

## 3. From the Fictional to the Real World: The Art-Disease Relationship

Illness and art have always been strictly bounded, especially when neurological and psychiatric diseases are involved [1]. Part of this interest is due to audience's speculations on a possible effect of the illness in providing inspiration to some human art ability, as writing and painting. Several well-known artists experienced migraine (e.g., Picasso, Lewis Carroll, and De Chirico) and some researchers think that their works could be in some way influenced by migraine features [2]. However, the role of migraine in the literary production by Lewis Carroll seems well defined [3].

The idea that Lewis Carroll (Charles Lutwidge Dodson's nom de plume) was a migraineur [3] is widely proven by his diaries. He often reported a “bilious headache” with severe vomiting, and in 1885 he wrote he “experienced, for the second time, that odd optical affection of seeing moving fortifications, followed by a headache” [4]. For this visual disturbance, in 1856, Carroll consulted an eminent ophthalmologist, William Bowman. He also published in his family journal a figurative representation of a person with the right half of the face, shoulder, and hand erased, compatible with a right paracentral negative scotoma, typical of migraine's visual aura (for a detailed image of the visual disturbance see Podoll and Robinson, 1999 [3]).

Lewis Carroll wrote two books dedicated to his heroine Alice, namely,* Alice's Adventures in Wonderland *(1865) and* Through the Looking*-*Glass, and What Alice Found There *(1871). In the story, Alice experienced several strange feelings, as slowing in time perception, while falling down the white rabbit's hole [5]:

*Either the well was very deep, or she fell very slowly, for she had plenty of time as she went down to look about her and to wonder what was going to happen next.*

In chapter II, her body shrank after drinking from a bottle labelled “DRINK ME”; then she ate a cake and became so large that she almost touched the ceiling. Examples of Alice experiencing partial macrosomatognosia and total microsomatognosia are, respectively, as follows:

*Curiouser and curiouser!' cried Alice; now I'm opening out like the largest telescope that ever was! Good-bye feet!*

* at first she thought it must be a walrus or hippopotamus, but then she remembered how small she was now, and she soon made out that it was only a mouse that had slipped in like herself.*

Later she experienced a feeling that could be identified as depersonalization:

*I almost think I can remember feeling a little different. But if I'm not the same, the next question is, who in the world am I?*

Also in* Through the Looking-Glass*, examples of time and space misperceptions are described in the form of feeling body acceleration without achieving distances. Such uncanny and disturbing descriptions, so unusual in children literature, may come from the direct experience of the author's own illness (for a pictorial representation of Alice changes you may see Sir John Tenniel's work on Project Gutenberg: http://www.gutenberg.org/files/114/114-h/114-h.htm).

## 4. Alice in Wonderland as a Medical Syndrome and a Classification Proposal

In 1952, Lippman firstly described several patients experiencing sensations of becoming remarkably tall or short during attacks of migraine. Lately, Coleman found the same symptoms in a young patient with schizophrenia, who referred that she felt just like Alice in Wonderland, because of her sensation of shrinking and enlarging (cited in Todd, 1955 [6]).

However, the first that used the term of Alice in Wonderland Syndrome (AIWS) was Todd, in 1955. He described six cases (four were migraineurs), reporting mainly somesthetic symptoms of feeling part of or the whole body as larger (macrosomatognosia) or smaller (microsomatognosia) than usual [6]. These symptoms were also associated with either visual illusions, including dysmetropsia, namely, macropsia and micropsia (objects or other people appearing bigger or smaller) and telopsia and pelopsia (objects or other people appearing respectively further or nearer), or disorders of consciousness, as feelings of derealization, depersonalization, somatopsychic duality (i.e., the idea to be split in two, more often vertically in the middle), and alteration in judgment of time. Some other symptoms that have been reported within the spectrum of AIWS are kinetopsia, auditory hallucinations and verbal illusions, hyperacusia/hypoacusia, dyschromatopsia, zoopsia, and complex visual hallucinations.

Since 1955, about 170 cases of AIWS have been reported in literature. To date, only part of them fits Todd's description, and therefore the inclusion in AIWS definition seems disputable for some of the reported cases. The lack of a standard classification yields a diagnostic issue regarding AIWS.

In attempt to reduce diagnostic puzzle, Podoll proposed considering somesthetic symptoms as the core of AIWS, while considering visual illusions and other complex psychic symptoms as facultative and not sufficient for the diagnosis by themselves [7].

Other authors differentiated patients in subgroups. Patients with somesthetic perceptual symptoms were classified as type A (about 9% of all cases), and cases with visual illusions alone were classified as type B (these were not described by Todd himself but paradoxically are the most prevalent type, counting up to 75%), while patients with coexistent somesthetic and visual symptoms are considered as type C (about 16% of cases) [8].

Recently, Liu et al. [9] proposed distinguishing type A AIWS patients from types B and C patients, who are the ones with extrapersonal dysperceptions, defining the latter two types as Alice in Wonderland-Like Syndrome (AIWLS). However, this decision appears to be quite artificial, since no pathophysiological reason supports this distinction.

In Table 1, we present a summary scheme of all the clinical features of Alice in Wonderland Syndrome, according to the abovementioned classification. We chose to include also visual illusions within the spectrum of AIWS, because of their high prevalence and frequent coexistence in literature with type A symptoms.

## 5. Epidemiology of AWS and Its Relation to Different Etiologies

The exact prevalence of the AIWS is unknown, for several reasons. First, no epidemiologic studies on large scale are available. Second, the lack of univocally accepted diagnostic criteria for AIWS casts a shadow on reported data that should then be considered carefully.

A study conducted on 3224 Japanese adolescents, aged 13 to 18 years, demonstrated that the occurrence of micropsia and macropsia was 6.5% in boys and 7.3% in girls, suggesting that the visual illusions of AIWS may be not so rare in general population [10].

The male/female ratio seems to vary with the age: while in younger age male are predominantly affected with 2.69-fold risk of having AIWS (in a sample aged 5 to 14 years) [9], no significant differences in sex prevalence were recorded in junior students (13–15 years old) by Abe et al. [10], while females were significantly more prevalent (56.7%) among senior students (16–18 years).

Out of 166 cases of AIWS published, the most common cause is migraine (27.1%), followed by infections (22.9%), principally EBV (15.7%). In decreasing order, other etiologies are as follows: brain lesions (7.8%), medicament (6%) and drugs (6%), psychiatric disorders (3.6%), epilepsy (3%), disease of the peripheral nervous system (1.2%), and others (3%). In about 20% of patients no cause of AIWS was found. In 65% of cases AIWS occurred in children under 18 years of age [11].

In a prospective study [12] on 9 children aged from 5 to 16 years, the mean age of AIWS onset was 8.5 years.

The following summarizes AIWS etiologies reported in literature. We chose to consider only cases with a diagnosis of AIWS, ignoring many other cases that could match the characteristic of AIWS but are not explicitly referring to it.

*More Common Causes Reported of AIWS*

*AIWS Etiologies*

Headaches
  - Migraine
  - Abdominal migraine
  - Cluster headache
  - Tension type headache
  - HANDL: syndrome of transient headache and neurological deficits with cerebrospinal fluid lymphocytosis
Epilepsy
  - Temporal lobe epilepsy
  - Frontal lobe epilepsy
Infectious diseases
  - Epstein-Barr virus
  - Coxsackie B1 virus
  - Cytomegalovirus
  - Influenza A virus
  - Mycoplasma
  - Varicella-zoster
  - Typhoid encephalopathy
  - Lyme neuroborreliosis
  - Streptococcus pyogenes (scarlet fever and tonsillopharyngitis)
  - Parainfective vasculitis
Cerebrovascular diseases
  - Intraparenchymal hemorrhagic stroke
  - Ischemic stroke
  - Cavernous angioma
  - Robin Hood syndrome
  - Pituitary infarction

Other organic brain diseases
  - Acute disseminated encephalomyelitis
  - Glioblastoma
Psychiatric disorders
  - Depressive disorder
  - Cotard Syndrome
  - Capgras Syndrome
  - Schizophrenia
  - Schizoaffective disorder
Drugs
  - Dextromethorphan
  - Cough syrup (containing dihydrocodeine and DL-methylephedrine)
  - Montelukast
  - Topiramate
  - LSD
  - Hallucinogen Persisting Perception Disorder (HPPD) after LSD withdrawal
  - Toluene-based solvent

## 6. Anatomical Correlation of AIWS

To date, only a few studies regarding AIWS reported neuroimaging evidences of damage at the level of cerebral cortices. A critical area for developing AIWS seems to be the carrefour of three major areas, the TPO-C [13], which is the crossroad of temporooccipital, parietooccipital, and temporoparietal junctions (TOJ, POJ, and TPJ). TPO-C is where visual and somatosensory information are integrated to generate the inner and external representation of self. This seems to be supported by several reports in which anatomical lesions of patients experiencing AIWS were located in right or left TPO-C. The spectrum of symptoms appears to be wider and more complex if lesions involve more anterior regions of the brain. In fact, theoretically, if a lesion is located in occipital regions we observe more likely simple visual disturbances, while if the lesion is closer to parietal and temporal areas also somatosensory and cognitive disorders will combine with the visual ones, resulting in more complex symptoms, as in an integration abnormality.

Some authors [14, 15] demonstrated that hemimicropsia can depend on a lesion in the inferior portion of the right parastriate area, sparing both the calcarine region and the geniculostriate projections, parts of Brodmann areas 18 and 19.

Hemimacropsia can result from lesions affecting the medial part of occipital lobe and the lingual and fusiform gyri, probably due to damage in the ventral portion of the occipitotemporal projections [16, 17], corresponding to the medial part of areas 17, 18, and 19 in Brodmann classification.

A SPECT study revealed hypoperfusion in the temporal-occipital lobe and perisylvian area during the acute stage of AIWS [18]. Interestingly, an fMRl study during an episode of micropsia in a child with AIWS revealed contemporary hypoactivation in occipital lobe and hyperactivation in the right superior parietal cortex. Moreover, in the same study, when a size comparison-based visual task was used in the same patient with micropsia, hyperactivation of right superior and bilateral inferior parietal cortices was found [13].

Body representation principally occurs in the primary sensory cortex (S1). In parietal cortex, the perceived representation of one's own body is rescaled through the integration of somatic signals from different body segments, as demonstrated by Ehrsson et al. [19]. In brief, authors induced a conflict between the real relative position of the waist and the hands of subjects, through tendons stimulation. Subjects' brain resolved this ambiguity recalibrating the perceived size of waist, resulting in partial microsomatognosia. The more intense patients' illusion was, the stronger the activity recorded by fMRI in postcentral and anterior intraparietal sulci was.

Other interesting pathophysiological data come from studies in which AIWS is associated with other neurological or psychiatric diseases. In a patient experiencing both dysmetropsia and Cotard's syndrome, a large cortical grey matter hyperintensity in T2 in the right temporal-parietal-occipital lobe with gyriform gadolinium enhancement on T1 was found in the acute phase and disappeared with the resolution of symptoms [20]. This association is worth analysing. and Cotard's syndrome is an extreme form of nihilism, characterized by the belief of being completely or partially dead. Recently a single-case PET study showed a globally decreased frontoparietal activity in Cotard's syndrome, but interestingly in this subject one major left parietal lesion was found on MRI [21].

A pathophysiological mechanism consistent with neuroimaging data here presented is that symptoms in AIWS may be caused by a dysfunction of the TPO-C, which is the brain area implicated in the integration of visual and somatosensory inputs. This may depend on either a direct functional alteration in brain areas forming the TPO-C (namely, TOJ, POJ, and TPJ) or an impairment of the primary visual sensory cortices, which favours an overrecruitment of parietotemporal associative areas implicated in active spatial attention tasks, with an unbalance among both visual system streams (this will be discussed in detail in the next paragraph).

However, other studies showed how AIWS could occur also when other brain areas outside the TPO-C are involved. SPECT during an episode of micropsia demonstrated hypoperfusion of frontal and frontoparietal areas [22]. In another case, hypoperfusion in the frontoparietal operculum caused micro- and macrosomatognosia and telopsia [23]. Another case of AIWS type B was attributed to a frontal lobe epilepsy seizure [24]. And, finally, a case of macro- and microsomatognosia was attributed to focal frontal lobe infarct in the superior right frontal gyrus and the genu of corpus callosum revealed by a CT [25].

We can speculate that frontoparietal connections are responsible for these cases. Corpus callosum contains fibers connecting parietal and frontal cortices involved in visual-motor tasks. Frontal lobes seem to play a top-down modulation on visual pathway, being the end of occipitotemporal stream involved in objects processing [26]. This feedback is strongly needed in resolving ambiguous visual perception. Sterzer and Kleinschmidt [27] demonstrated that inferential processing by frontal lobe is stronger when sensory input is conflicting and slightest when it is almost unambiguous. Therefore, the impairment in this inferential process of visual information in frontal lobe can contribute to overrecruitment of associative areas, probably producing the AIWS symptoms as well as primary visual cortex deficiencies.

## 7. AIWS as an Alteration of Visual and Somatosensory Integration: A Model Proposal

The clinical spectrum of symptoms attributable to AIWS, especially if we include types A, B, and C in the definition of this syndrome, is extremely wide. This is not surprising considering the high-level integration of sensory information occurring in TPO-C. Here we review physiological mechanism of such integration, whose alteration may be responsible for AIWS. In an elegant study that investigated brain activity during out-of-body experiences in 5 patients (four with epilepsy and 1 with familial hemiplegic migraine), TPJ and the angular gyrus were found involved in all subjects, with no side preference [28]. Not surprisingly, TPJ is involved in visuospatial neglect [29] and in egocentric perspective changes in healthy subjects [30].

Below, we will discuss separately the involvement of image-forming and non-image-forming visual pathways in the integration of multimodal sensory information for the development of a conscious perception.

### 7.1. Image-Forming Visual Pathway

Visual pathway has been considered as divided into two streams: dorsal (or parietal) stream and temporal (or ventral) stream. The parietal stream is responsible for objects' movement and position, while the temporal stream encodes object's size, form and colour [31].

Intuitively, successful arm-reaching and grasping action should rely on both arm position and objects distance and dimension. Milner and Goodale [32] demonstrated that parietal stream is implicated in visuomotor tasks, relying on the absolute size of objects. Thus, goal-directed perception is not endangered by size-contrast illusions. Conversely, temporal stream perceives objects into scene-based frame, rescaling them to relative size and distance. In this view, type B AIWS symptoms, such as micropsia and macropsia, should be largely but not only ascribed to the temporal lobe.

Although this could suggest that dorsal stream is mostly implied in judging size and distance of objects reachable by hand, while temporal stream is mostly implied in the judgment of unreachable objects, near and far space can be remapped by purposeful use of tools that are considered by our brain as own arm extension [33].

In far space perception Chen et al. [34] demonstrated higher activity in the lateral occipital cortex, consistently with an involvement of the ventral stream. By contrast, an activation of superior occipital sulci crossing the cuneus was found in near-space judgments, suggesting an involvement of the dorsal stream.

However, the same group of authors showed that near-space versus far space perception can be influenced by subject's intentions, namely, the egocentric or allocentric judgment. In fact, we can estimate the position and size of a far object by activating the dorsal stream, instead of ventral stream, if the object is relevant for our purposes or manipulable or if object's manipulation can increase our ability to explore and understand the object itself, through a visual-motor schema (egocentric judgment). By contrast we can consider near and reachable objects as unrelated to us if not interesting, assessing their position and size relative to other objects (allocentric judgment). Interestingly, the mostly activated area in these contrasting situations (i.e., when we apply an egocentric reference to far objects or an allocentric reference to close ones) is POJ, suggesting that this area, within the TPO-C, is implied in the exchange of information between the ventral and dorsal stream and, therefore, may explain the cooccurrence of type A and type B symptoms.

Complete AIWS forms might result from the impairment of areas involved in visual-somesthetic integration, inside and outside POJ and TPO-C, whose aim is to achieve a correct relationship between internal and external space. Lesions far from these areas can solely lead to partial alteration of one or the other, while lesions closer to the high-level integration region (i.e., TPO-C) may produce symptoms including both of these sensory modalities. The wide spectrum of clinical manifestation can be then a continuum from simple to complex symptoms.

### 7.2. Non-Image-Forming Visual Pathway

Another possible explanation for developing AIWS is the involvement of the non-image-forming visual pathway. Indeed, the non-image-forming visual pathway largely influences the integration activity between visual and temporoparietal cortices. The non-image-forming visual pathway is a secondary visual system present in mammalian, which is responsible along with the image-forming visual system for physiological responses, as circadian photoentrainment and pupillary light reflex, as well as pathological ones, as photophobia [35, 36].

In brief, the non-image-forming visual pathway is principally supported by melanopsin-containing intrinsically photosensitive retinal ganglion cells (ipRGC) in the retina that project through the optic nerve to several regions outside the normal image-forming visual system (optic nerve → lateral geniculate → visual cortex) [35–37].

Non-image-forming visual system projects to the suprachiasmatic nucleus (SCN), intergeniculate leaflet (IGL), the habenula, the pineal, and olivary pretectal nucleus (OPT) and directly connects retinal ganglion cells with the pulvinar within posterior thalamic nuclei, via optic chiasm. From the pulvinar it leads to the associative cortices, including primary visual areas (Brodmann area 17), secondary visual areas (Brodmann areas 18 and 19), visual inferotemporal areas (Brodmann area 20), posterior parietal associative areas (Brodmann area 7), and frontal eye fields and prefrontal areas.

Though less known, this system offers several hints to physiological speculation on the role of thalamus as regulator of information flow between high-level associative cortices. We know of a case of spatial hyperschematia, compatible with AIWS symptoms, in a patient with a lesion disconnecting the thalamus from right parietal and temporal cerebral cortices as well as the insula [38].

The connections between visual, parietal, and temporal cortices in the image- and non-image-forming visual system may be more complex than expected and, in some cases, it may pass through the thalamus [39]. The data by Imbert and Bignall showed that visual stimulation can activate the orbitofrontal cortices without activating the somatosensory cortex in a model of thalamectomized cat [40]. It suggests that a relevant part of projections from the occipital cortex passes through the thalamus to be conveyed to the somatosensory cortex. Interestingly, in this study, the lateral geniculate nucleus was intact, showing that it is not an obligatory relay for visuosomatosensory connections. In support of these data, in a recent case of facial blind sight, some connections between the lateral fusiform gyrus and thalamic nuclei have been implicated in unconscious residual visual faculties of the subjects [41].

Although the data available at present do not allow supporting an integrative theory between the interplay of image-forming and non-image-forming visual systems, we believe that an unbalanced information flow between cortical areas is fundamental for developing AIWS. Due to its role of orchestra director in integrating different sensory modalities in a unique, multimodal perception, the thalamus may be highly implicated in AIWS. This is not surprising if we keep in mind that bimodal stimuli (visual plus tactile) may activate somatosensory regions more than unimodal stimulation [42]. Although multisensory interplay has always been considered occurring in superior colliculus, some evidence showed that specific thalamic areas (mainly the pulvinar) have integrating functions of sensory modalities [43].

## 8. AIWS and Migraine

Although AIWS is not enlisted in the International Classification of Headache Disorders (ICHD) 3 beta, even if it seems strictly related to migraine, in fact, migraine is the first cause of AIWS in adults (27.6%) and the second in children (26.8%). Recently, Valença et al. [44] proposed diagnostic criteria for migraine-associated AIWS that could be provisionally applied to migraineurs while waiting for a general classification as follows.

*Diagnostic Criteria for Migraine-Related AIWS Proposal by Valença et al. [44]*

- One or more episodes of self-experienced body schema illusion or metamorphopsia
- Duration < 30 min
- Accompanied by headache or a history of migraine
- RMI, CSF, and EEG all normal (visual evoked potentials may be abnormal)

Interestingly, several children patients, experiencing AIWS for etiologies other than migraine, had a relevant family history of migraine and had or lately developed migraine headache. This result is confirmed by a study on young students with visual illusions [10]: one-third of subjects with episodic illusions were probable migraineurs.

In literature, many authors reported a temporal relation between migraine and AIWS. Smith et al. [12] presented a 1-year follow-up of 9 children. They found that children developing both migraine and AIWS were, on average, 8.4 years old at AIWS onset and about a year older at the headache onset. In 4 of these children AIWS occurred before the headache, in 3 it occurred after the headache, and in 2 AIWS and headache coexisted. Weidenfeld and Borusiak [45] mentioned a 7-year-old boy who, 3 years after episodes of micropsia, macropsia, and pelopsia, developed a tension type headache. Liu et al. [9] reported that 27% of patients with AIWS (regardless of the etiological cause) became migraineurs during the follow-up period, suggesting that AIWS may be a type of migraine aura or a migraine equivalent.

From the pathophysiological point of view, the association between migraine and AIWS may be explained by the neuronal effect of cortical spreading depression (CSD) on the abovementioned brain areas. AIWS is more frequent in children and it should depend on structural differences between children's and adult's brain: according to Flechsig's order of myelination of cortical areas, associative ones are the last to develop. Thus, the immaturity of associative areas could explain how these are more vulnerable to spreading depression than in adult equally affected by migraine. To date, the close association between migraine and AIWS supports this idea, but at present this remains a speculation.

Although a detailed description of migraine pathophysiology lies outside of our topic, we will give a brief insight of migraine mechanisms in order to clarify the relationship between AIWS and migraine.

Migraine is a recurrent neurological disorder characterized by phases of well-being (interictal phase) and painful attacks (ictal phase). However, the main pathophysiological alterations are present in both interictal and ictal phase. Out of a migraine attack, migrainous brain appears hyperresponsive to repeated sensory stimuli (e.g., visual, somatosensory, and auditory) without the normal ability to habituate to reduce the neural workload according to stimulation saliency and metabolic supply. This neurophysiological pattern, described in many experiments, is called deficit of habituation theory (for a review, see Magis et al. [46]). Conversely, just before and during a migraine attack, brain habituation changes over time and finally appears restored in the attack phase.

Also, in chronic migraine (more than 15 days of headache monthly), a normal habituation is found even in the rare well-being phases (interictally), suggesting that, in these forms, migraineurs brain is stuck in a sort of “never-ending attack” even outside the ictal phase [47]. As a matter of fact, habituation deficit appeared steeper in severe condition and normalizes with successful therapy [48–50].

The lack of habituation is an epiphenomenon recorded at the level of cerebral cortices due to an abnormal thalamocortical regulation dysfunction, called thalamocortical dysrhythmia. This abnormality, found in several neurological and psychiatric diseases [51], seems in fact responsible for the altered cortical sensory information processing found in migraine brain [52] and probably also implicated in the cyclical rhythm between ictal and interictal periods.

Ictally, cortical-related phenomena are due to CSD, which is responsible also for migraine aura [53]. Cortical spreading depression is a traveling depolarization wave, moving through cerebral cortices at a rate of 3–5 mm/min, with a brief neuronal excitation wavefront, followed by a prolonged inhibition of neuronal activity. CSD moves through the cerebral cortices regardless of the functional division and vascular territories, so that the succession of aura symptoms may be different from an attack to another or inconstant. Some authors speculated that CSD might trigger a migraine attack also in form of migraine without aura triggering downstream nociceptive pathways [54–56].

In a study on patients with migraine with complex aura (comprising not only visual but also sensory and language dysfunctions), a higher level of dysfunction in comparison to patients with simple aura, correlating higher interictal abnormalities with more complex aura pattern CSD, was found [57]. Also, increased VEP amplitude was correlated with a longer distance in the spreading of paroxysmal EEG responses [58]. Interestingly, it seems to depend on an increased functional connectivity in wide neural networks between occipital and parietotemporofrontal areas that are principally regulated by the activity of the thalamus [59–61].

Regarding AIWS, not only are complex forms of aura related to a higher level of dysexcitability and larger areas involved by CSD, but also some studies on palinopsia disturbances suggested that diffuse, persistent alterations of neuronal excitability, also described during the interictal period [46], might be responsible for impairing light or motion processing, causing a large spectrum of visual symptoms including dysmetropsia [62]. A case of spatial hyperschematia without spatial neglect was described after an ischemic lesion causing a disconnection between the right insula and the posterior part/pulvinar of the thalamus [38]. In this subject, an extension of peripersonal and extrapersonal space, especially from the egocentric point of view, was observed. Namely, thalamoparietal projections are thought to be implicated in the perception of egocentric space [63]. Besides the role of thalamus in controlling the flow of sensory information to cerebral cortices, a study showed that CSD might activate the thalamic reticular nuclei [64].

## 9. AIWS and Infections

Although the most frequent cause of AIWS, especially in children, is acute infections, the pathophysiological mechanism of AIWS during such episodes remains highly speculative. Moreover, in the report by Weidenfeld and Borusiak [45], a large part of patients experiencing AIWS during infectious disease were lately diagnosed with headache, so we cannot exclude that also in these patients AIWS was a sort of migraine equivalent.

As previously discussed, in a child experiencing AIWS during EBV infections, an unbalance in cortical activation between low and high order cortices in TPO was described [13]. Even though pathophysiological data are scarce, on theoretical basis we can reason that the unbalance between primary and secondary cortices may be attributed to a local irritative activity produced by infections, with possible developing of electrical phenomena, as epileptiform seizures and/or CSD, as suggested by reports [65] in which local occipitoparietal intermittent slowing of EEG was found in patients with AIWS, together with CSF leukocytosis, during the acute phase of the disturbance.

Epileptiform activity and CSD may be induced by similar mechanisms: hypoxia, hypoglycemia, high concentrations of K^+^, or Na^+^/K^+^ pump inhibition [66]. In experimental models, changing the level of potassium in the bath can produce spontaneous periodic seizures when K^+^ reaches high level (about the double of normal concentration), while CSD is evoked when potassium elevates over 8-fold the normal concentration. Hypoxia as well as high concentration of K^+^ or inhibition of Na^+^/K^+^ pump is common in neural inflammatory injuries [67].

## 10. Conclusions and Future Perspectives

AIWS is still poorly known and probably misdiagnosed for the lack of clear and universally accepted diagnostic criteria. In migraine-related AIWS, which probably represent the most common form, a practical diagnostic proposal has been recently published [44].

To date, there is no agreement on what is and what is not AIWS, so some authors [11] include as part of AIWS symptoms such as dyschromatopsia, entomopia, mosaic vision, palinopsia, polyopia, visual perseveration, visual allachesthesia, and prosopometamorphopsia.

We acknowledge that clinical presentation of AIWS may be complex and may vary according to the different pathophysiological mechanisms. In migraine, which is highly related to AIWS and possibly a migraine equivalent [44], the plethora of symptoms may depend on activation of areas contiguous to TPO-C but not involved in the same neural circuit (i.e., face recognition, colour perception, etc.) by CSD.

Although only a minority of AIWS cases reported specific information on site of brain correlate, it appears consistent that brain alterations responsible for AIWS are located in TPO-C, where the dorsal and ventral streams of visual system are integrated with somatosensory and vestibular inputs. Key areas in AIWS may be POJ and angular gyrus. POJ area is mostly activated in unconventional spatial cognition, as imagining grabbing something far from us or ignoring something close to us, namely, in the shift from egocentric to allocentric judgment from far to near objects. On the other hand, angular gyrus was found activated in all patients experiencing complex sensory disturbances during out-of-body perception [28].

While type B symptoms can be distinguished on the basis of dorsal/ventral stream division, complete AIWS presentation seems to rely on alteration of key brain areas, in which space and body representation are integrated, as associative areas of TPO-C. Also, subcortical regions, and especially the thalamus, may be implicated in the development of AIWS, due to its role in coordinating sensory information flow and integration. In our opinion better anatomical and functional studies of AIWS pathophysiological mechanisms are eagerly needed, but a main prerequisite is a clear and univocally accepted classification based on precise diagnostic criteria.

## Figures and Tables

**Table 1:** Summary of proposal classification of symptoms.

Types	Obligatory symptoms	Facultative symptoms
Type A	Aschematia: partial or total macrosomatognosia or microsomatognosia; paraschematia	Derealization, depersonalization, somatopsychic duality, aberration in judgement of time
Type B	Macro- and micropsia and/or tele- and pelopsia. When micropsia and telopsia appear at the same time and for the same object: porropsia Lilliputianism (people appearing smaller)	
Type C	Type A + type B symptoms	
